# Secure hardware IP of GLRT cascade using color interval graph based embedded fingerprint for ECG detector

**DOI:** 10.1038/s41598-024-63533-7

**Published:** 2024-06-10

**Authors:** Anirban Sengupta, Aditya Anshul

**Affiliations:** Indian Institute of Technology (IIT) Indore, Indore, India

**Keywords:** Secure hardware IP, GLRT cascade, Fingerprint biometric, ECG, CIG, Engineering, Electrical and electronic engineering

## Abstract

This paper presents a security aware design methodology to design secure generalized likelihood ratio test (GLRT) hardware intellectual property (IP) core for electrocardiogram (ECG) detector against IP piracy and fraudulent claim of IP ownership threats. Integrating authentic (secure version) GLRT hardware IP core in the system-on-chip (SoC) of ECG detectors is paramount for reliable operation and estimation of ECG parametric data, such as Q wave, R wave and S wave (QRS) complex detection. A pirated GLRT hardware IP integrated into an ECG detector may result in an unreliable/erratic estimation of ECG parametric data that can be hazardous and fatal for the end patient. The proposed methodology presents an integrated design flow to secure micro GLRT and GLRT cascade hardware IP cores for the ECG detector, using the colored interval graph (CIG) framework based fingerprint biometric, during high level synthesis (HLS). The proposed approach integrates a fingerprint biometric based security constraint generation process for securing the GLRT hardware IP core. This paper also presents a secure register transfer level (RTL) datapath design corresponding to micro GLRT and GLRT cascade hardware IP cores with embedded IP vendor's fingerprint. The proposed secure GLRT hardware IP core embedded with fingerprint biometric achieves superior results in terms of probability of coincidence and tamper tolerance than other security approaches. More explicitly, the proposed approach reports a significantly lower value of probability of coincidence and stronger value for tamper tolerance. Further, the proposed approach incurs zero design cost overhead.

## Introduction

Accurate detection of cardiovascular diseases or disorders (CVDs) has become primarily important because of the rise in the cardiac failure rate and various cardiovascular disorders such as arrhythmias, atrial enlargement, Wolff–Parkinson–white syndrome, myocardial ischemia, ventricular hypertrophy, heart failure, hypertrophic cardiomyopathy long QT syndrome, etc.^1,2^. It is essential to accurately diagnose these CVDs within an appropriate timeline to ensure their proper remedy. Subsequently, an early and accurate diagnosis also helps in taking precautionary steps to avoid any future greater complications. ECG detector is a widely used application/device which is used to study the condition of a person's (or patient's) heart by capturing different signals generated by the heart, using different electrodes. Further, the waves generated and recorded by an ECG device are analyzed by health experts (especially a doctor) to draw a conclusion regarding the patient's heart condition. ECG detector is also a vital component in cardiac pacemaker devices. ECG detector comprises of various critical components that perform crucial functions such as GLRT unit, filtering unit, analog to digital converter unit, etc.^3^. GLRT unit is the primary component of the ECG detector that executes computation-intensive functions to estimate heart rate using (Q wave, R wave and S wave) QRS wave complex from incoming filter signals. Therefore, it is essential to design the GLRT unit of the ECG detector as a reusable hardware IP core because of its wide usability and computation-intensive nature. The high level synthesis (HLS) framework of the hardware design process facilitates designing the GLRT unit as a dedicated reusable hardware IP core (in register transfer level (RTL) form)^4,5^.

From the perspective of the end user (patient), the safe and reliable functioning of the GLRT unit in the ECG detector is critical as it is responsible for the generation of important ECG parametric data such as Heart Rate (HR), PR Interval (PRI), QRS Interval (QRSI), QT Interval (QTI), QTC Interval (QTCI). For example, Table 1 reports the acquired ECG data of patient X from the department of non-invasive cardiology of cardiology research laboratory. The data has been recovered ethically with the consent of the respective patient. It depicts the normal ECG parameter range^6–9^ and the acquired values. Designing a secure version of GLRT hardware IP core ensures the integration of authentic designs into the system-on-chip (SoC) of ECG detectors. A pirated GLRT hardware IP core integrated into an ECG detector can cause hazardous consequences for a patient. This is because an ECG detector SoC involves design process through multiple untrustworthy offshore entities in the global supply chain, which increases the risk of IP piracy^10,11^. A pirated (i.e., counterfeited) GLRT hardware IP core is unreliable and may contain malicious logic that could result into inaccurate detection of vital ECG parametric data, erratic behavior or functionality of the ECG detector, mistimed pulse from ECG detector for cardiac pacemaker devices. Further, since GLRT units are vital for cardiac pacemakers, which are implanted in a patient's heart surgically for a long period (more than eight years), therefore its secure hardware IP core versions used for integration in cardiac pacemakers are imperative. Moreover, an adversary can also make a false/fraudulent claim of ownership of the designed GLRT hardware IP core.

**Table 1:** Acquired ECG data of patient X, is age: 69Y 6 M 3D, gender: male, report date: 05/may/2023 04:27 pm from department of non-invasive cardiology of cardiology research laboratory.

ECG parameters	Normal ECG parameter range		Acquired ECG data	
	Parameter name	Value	Parameter name	Value
Heart rate (HR)	HR	60–100 bpm	HR	75 bpm
PR interval (PRI)	PRI	0.1 s–0.2 s	PRI	0.138 s
QRS interval (QRSI)	QRSI	0.07 s–0.10 s	QRSI	0.072 s
QT interval (QTI)	QTI	0.36 s–0.44 s	QTI	0.34 s
QTC interval (QTCI)	QTCI	0.36 s–0.44 s	QTCI	0.382 s

Significance of abnormal range (> upper limit): Cardiovascular disorder such as arrhythmias, atrial enlargement, Wolff–Parkinson–white syndrome), myocardial ischemia, ventricular hypertrophy, heart failure, hypertrophic cardiomyopathy long QT syndrome, etc.

This paper presents a novel secure hardware IP of GLRT cascade using color interval graph (CIG) based embedded fingerprint, for ECG detector. The proposed approach discusses designing GLRT micro and GLRT cascade hardware IP core for ECG detectors for the first time in literature. The proposed approach harnesses the power of fingerprint biometrics to generate a fingerprint-based digital template. Further, the generated digital template is used to determine the secret hardware security constraints, which are embedded in the design of GLRT hardware IP core using the CIG framework of the HLS process. The presence of fingerprint-based unique digital evidence inside the GLRT hardware IP design facilitates distinguishing and isolating authentic GLRT hardware IPs from pirated ones before integration in ECG detectors.

## The novel contributions of the paper are as follows:

- Presents the design methodology of GLRT hardware IP core for ECG detector for the first time in the literature.
- Presents a secure GLRT hardware IP core for ECG detector using fingerprint biometric-based hardware security methodology during HLS.
- Presents CIG framework and RTL datapath of a secure GLRT hardware micro IP core and secure GLRT hardware cascade IP core.

The rest of the paper is as follows. “Related work” provides insight into the related approaches. “Overview of ECG detector” highlights the overview and importance of ECG detectors. “Threat model and overview of the proposed methodology” discusses threat model and overview of the proposed methodology. Further, a detailed discussion of the proposed methodology is presented in “Details of the proposed methodology”. The important findings of proposed methodology and discussion on various attack scenarios have been reported in “Results and analysis”. Finally, “Conclusion” concludes the paper.

## Related work

Few works in the literature so far have discussed designing area and power-efficient health monitoring devices, such as ECG detectors. Authors in^3^ present a high level transformations (HLTs) based design of ECG detector. Authors in^3^ have used folding and cutset retiming (CTR) HLTs on the GLRT architecture of the ECG detector to obtain an area-efficient ECG hardware design. Folding helps in reducing the multipliers, adders, and delay elements, while CTR helps in reducing the architecture's clock period by altering the delay element's position in the critical path. Authors performed FPGA synthesis of the modified architecture to execute the testing process^3^. The presented approach in^3^ facilitates the generation of the area and power-efficient design of ECG detectors using folding and cutset retiming. However, it is incapable to design secure RTL datapath of micro GLRT and GLRT cascade macro hardware IP core for ECG detector using the HLS framework, having immunity against IP piracy and fraudulent claim of IP ownership threats, like in the proposed approach. Further, an estimation methodology between good and bad ECG signals from long-term captured ECG data of wearable devices is presented in^12^. The authors in^12^ have discussed a methodology to classify the incoming ECG signals into three classes, i.e., signal worthy of full-wave analysis, only QRS complex detection, and unwanted signals using signal-to-noise (SNR) ratio curve computation. The presented ECG signal classification approach facilitates easier and more accurate classification of long-term collected ECG data. It saves ECG data analysis time by categorizing the data as useful or unreliable. Next, authors in^13^ discuss efficient hardware of ECG devices with a low-frequency artifact (LFA) reduction approach. LFA present in ECG signals increases the ECG interpretation complexity. The presented LFA reduction approach for ECG detector hardware devices in^13^ provides a significant improvement over traditional reduction techniques with lower power consumption and generation of high-quality signals. References^12^ and^13^ focus on designing low-power ECG detectors with increased accuracy. However, Refs.^12,13^ does not provide security of micro GLRT and GLRT cascade macro hardware IP for ECG detector against IP piracy, fraudulent claim of IP ownership threat, tampering using brute force attack, and false positives. Additionally, Ref.^14^ discusses an efficient and power-saving technique to generate low-power hardware for complex applications useful in remote health monitoring systems using parallelization and optimization techniques. Authors in^14^ focus on developing domain-specific hardware accelerators with reduced energy consumption in biomedical applications. In contrast, the proposed methodology presents an integrated HLS-based design framework to generate a secure micro GLRT and GLRT cascade hardware IP core for ECG detectors. As discussed in the introduction, GLRT is the core component of the ECG detector that facilitates several crucial tasks, including complex R-wave detection by performing computation-intensive functions. Therefore, developing a secure GLRT hardware IP core for ECG detectors is crucial from the perspective of safe and reliable estimation of ECG parameters.

Further, Refs.^15–19^, and Ref.^20^ are some of the major state-of-the-art hardware security approaches presented in the literature. Hardware watermarking^15–17^ are the most prominent approaches in the field of hardware security, which employs encoding variables (i.e., digit encoding), signature size, and its combination as primary components to secure the hardware devices. Hardware watermarking discussed in^15^ and^17^ uses binary variable watermarking and quadruple variable watermarking to secure digital signal processors (DSP) hardware IP cores. Further, Ref.^16^ harnesses the power of mathematical relations to employ hardware watermarking. Reference^16^ generates relations between input data, initial values of internal computations, and output data to generate the final watermark. Reference^17^ supersedes^15^ and^16^ in terms of security robustness due to the generation of larger security constraints resulting into lower probability of coincidence and stronger tamper tolerance. However, the discussed approaches^15,16^, and^17^ do not address the security of GLRT hardware IP cores against IP piracy, fraudulent claim of IP ownership threat, tampering using brute force attack and false positives. These approaches provide weaker security than fingerprint biometric (employed in the proposed approach) in the context of IP protection due to chances of compromise in digit encoding and signature combination. Subsequently, Ref.^18^ presents a signature-free steganography-based security approach, where the register allocation information and threshold entropy value are used to generate security constraints. However, a compromised threshold entropy makes^18^ vulnerable to piracy attacks. Further, Ref.^19^ presents a digital signature-based hardware security approach to secure DSP applications. Reference^19^ harness the power of RSA cryptosystem and SHA-512 hash digest computation to generate the security constraints. Ref.^19^ becomes vulnerable if the RSA key gets leaked, besides generating limited security constraints. Approaches Refs.^18^ and^19^ are not capable to provide robust security against tampering attack, ghost insertion search attack, brute-force attack, and forgery attack, unlike the proposed approach which is resilient against the aforesaid threats. This is because the proposed approach exploits biometric fingerprint of IP vendor to generate and embed watermark into the IP design as a detective security countermeasure. Involvement of several security variables such as (a) number and type of minutiae points, (b) coordinates of minutiae points as a function of its position and ridge angle, (c) concatenation order of minutiae points, and (d) mapping/embedding rules, makes the proposed approach more robust against the standard attacks. Further, Refs.^18,19^ does not secure GLRT micro IP and GLRT cascade against IP piracy and false IP ownership. Additionally, Ref.^20^ discusses an encrypted signature-based approach using AES-128 and MD-5 cryptosystems to secure hardware systems. Furthermore, authors in^21,22^ have proposed multi-variable signature encoding based fingerprint during HLS for IP protection from the IP buyer’s perspective. Approaches Refs.^21^ and^22^ use crypto-based multivariable fingerprinting and multivariate signature encoded fingerprinting compared to biometric fingerprinting used in the proposed approach. Further, approaches Refs.^21^ and^22^ do not target security of GLRT IP used in medical applications such as ECG detectors, unlike the proposed approach. Finally, the hardware security strength of^21^ and^22^ is weaker than the proposed approach in terms of probability of coincidence and tamper tolerance ability.

All above discussed security approach does not present an integrated design flow to generate a secure GLRT hardware IP cores RTL design, besides providing weaker security due to the generation of limited security constraints and vulnerability of the security scheme. In contrast, the proposed methodology employs CIG based fingerprint biometric driven hardware security methodology to secure the GLRT hardware IP core design for the ECG detector. The fingerprint biometric security methodology provides more robust security in terms of lower probability of coincidence and higher tamper tolerance.

Further, physical unclonable functions (PUFs) have been used to ensure hardware security^23–25^ by assigning a distinct signature to each manufactured/fabricated chip, leveraging the inherent random process variations that arise during the manufacturing process. This unique signature serves as a fundamental means of identifying counterfeit or illicitly fabricated chips. Notably, PUFs have found applications in protecting FPGA-based intellectual properties (IPs) against issues such as overproduction and overuse that also arise at the foundry level^23^. While PUFs excel in the domain of device authentication/checking overproduction, hardware watermarking approaches take a different trajectory, primarily focusing on detecting IP piracy (of soft IPs) and detecting false claim of IP ownership (rather than targeting to prevent chip overproduction). The proposed fingerprint biometric-based watermark is covertly embedded as the IP vendor's digital evidence and diffused (scattered) throughout the IP design. The embedded watermark is used to verify the authentic IP design (GLRT core) or provide detective control in case of an IP piracy, besides nullifying ownership conflict. However, PUF-based techniques cannot be used to detect piracy of soft IPs (as a detective countermeasure) and induce detective control of pirated IP cores.

It has been clearly established in the literature^26,27^, that hardware security methods can be classified into two categories: (a) detective security countermeasure and (b) preventive security countermeasure. Logic locking provides preventive control against reverse engineering threat, while hardware watermarking provides detective control against IP piracy and false IP ownership claim. Logic locking aims to provide logic encryption of netlist at the gate level with the help of keys. Here, the main goal is to thwart reverse engineering attacks as a preventive countermeasure. However, logic locking techniques^28,29^, may incur gate overhead due to addition of locking gates into the IP design. Further, logic locking techniques are subjected to SAT attack, removal attack and key-sensitization attacks besides side channel attacks due to involvement of secret keys. On the contrary, the proposed approach is resilient against SAT attacks, removal attacks and key-sensitization attacks as it does not use keys to encrypt a gate-level IP design. Rather, the proposed approach aims to embed invisible, imperceptible biometric watermark during HLS of the IP design to provide detective countermeasure against IP piracy and nullify ownership conflict.

### Approval for human experiments

We confirm that all methods were carried out in accordance with relevant guidelines and regulations. We confirm that all experimental protocols were approved by Indian Institute of Technology Indore. We confirm that informed consent was obtained from all subjects and/or their legal guardian(s).

## Overview of ECG detector

**Figure 1:**
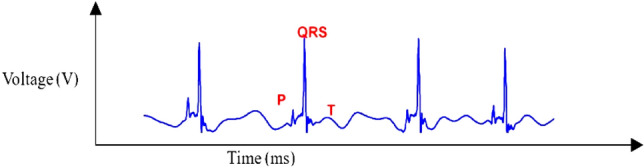
ECG wave recorded through electrode for reference; PR interval—duration between onset of atrial depolarization and ventricular depolarization; QT interval—duration between onset of ventricular depolarization and end of ventricular repolarization.

The ECG detector comprises of a filtering unit, noise detector, GLRT cascade unit, summer and threshold processing unit. ECG detector is used for the detection of ECG parametric data for heart activity evaluation based on the captured heart signals using electrode leads. The filtering unit in the ECG detector is responsible for generating monophasic (pulse of energy in a single direction) and biphasic (pulse of energy in two directions) outputs. These filtered outputs are fed as input to the GLRT unit to generate the presence of R wave. The outputs of GLRT cascades are summed and compared using a threshold processing function to differentiate cardiac signals from noise signals. Noise signals such as muscle artefact (noise signals generated due to muscle movement), power line interference, baseline wander, and instrumentation noise^30,31^ are common while recording heart signals. Figure 1 depicts an example of an ECG waveform recorded through the electrodes. As shown in Fig. 1, the ECG waveform consists of three segments P-wave, QRS-complex (Q, R, and S wave) and T-wave. The different waves and their corresponding intervals are depicted in Fig. 1 and Table 1, respectively. For example, any immediate upward deflecting wave after a P wave is an R wave, and a downward deflecting wave after a P wave is a Q wave^32^. Similarly, the PR interval shows the duration between the onset of atrial depolarization and ventricular depolarization; and the QT interval shows the duration between the onset of ventricular depolarization and the end of ventricular repolarization. The accurate detection of the QRS complex is challenging. More explicitly, accurate R wave estimation is challenging due to time-varying behavior and physiological variation^33^. The GLRT unit is the core of the ECG detector that facilitates the accurate detection and estimation of QRS complex or R wave. Further, as discussed in the introduction, ECG is widely used for detecting various CVDs and is an important component of cardiac pacemakers.

### GLRT overview

**Figure 2:**
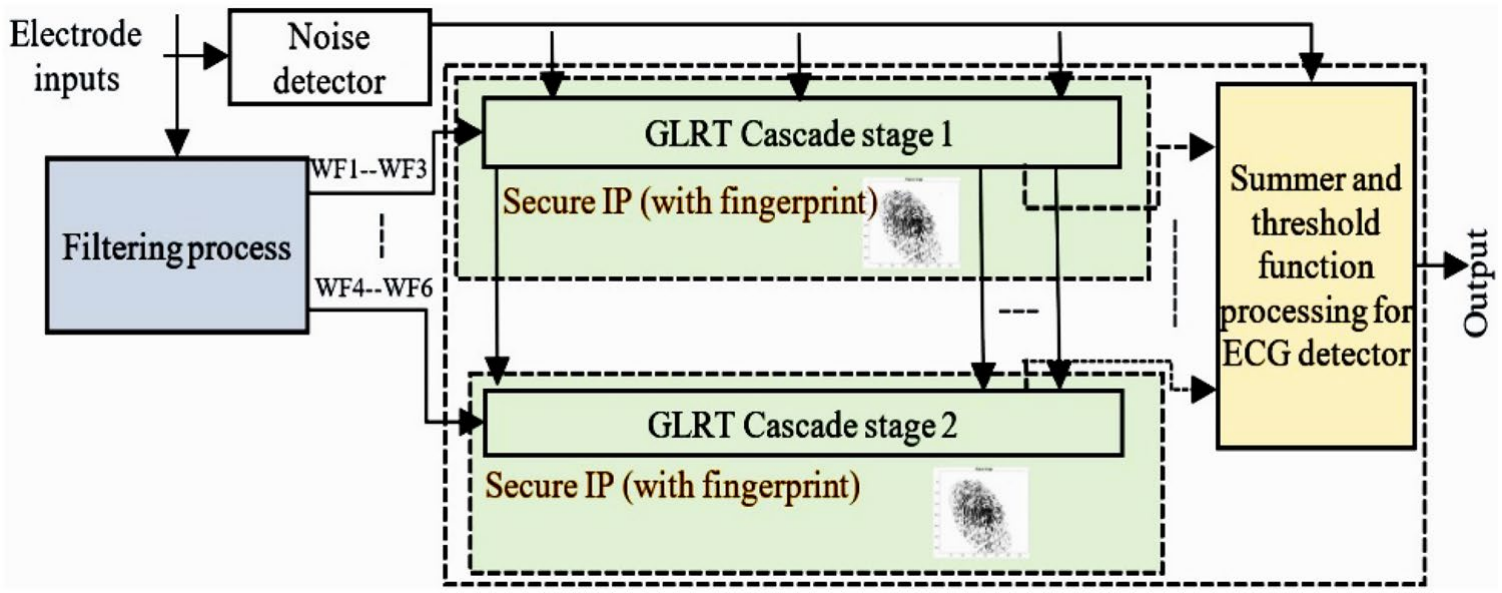
Proposed secure hardware IP of GLRT cascade for ECG detector.

**Figure 3:**
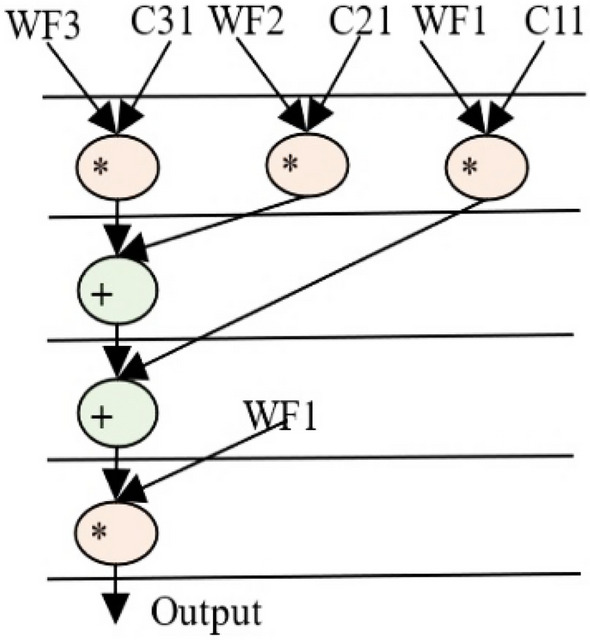
GLRT DFG of proposed micro IP.

**Figure 4:**
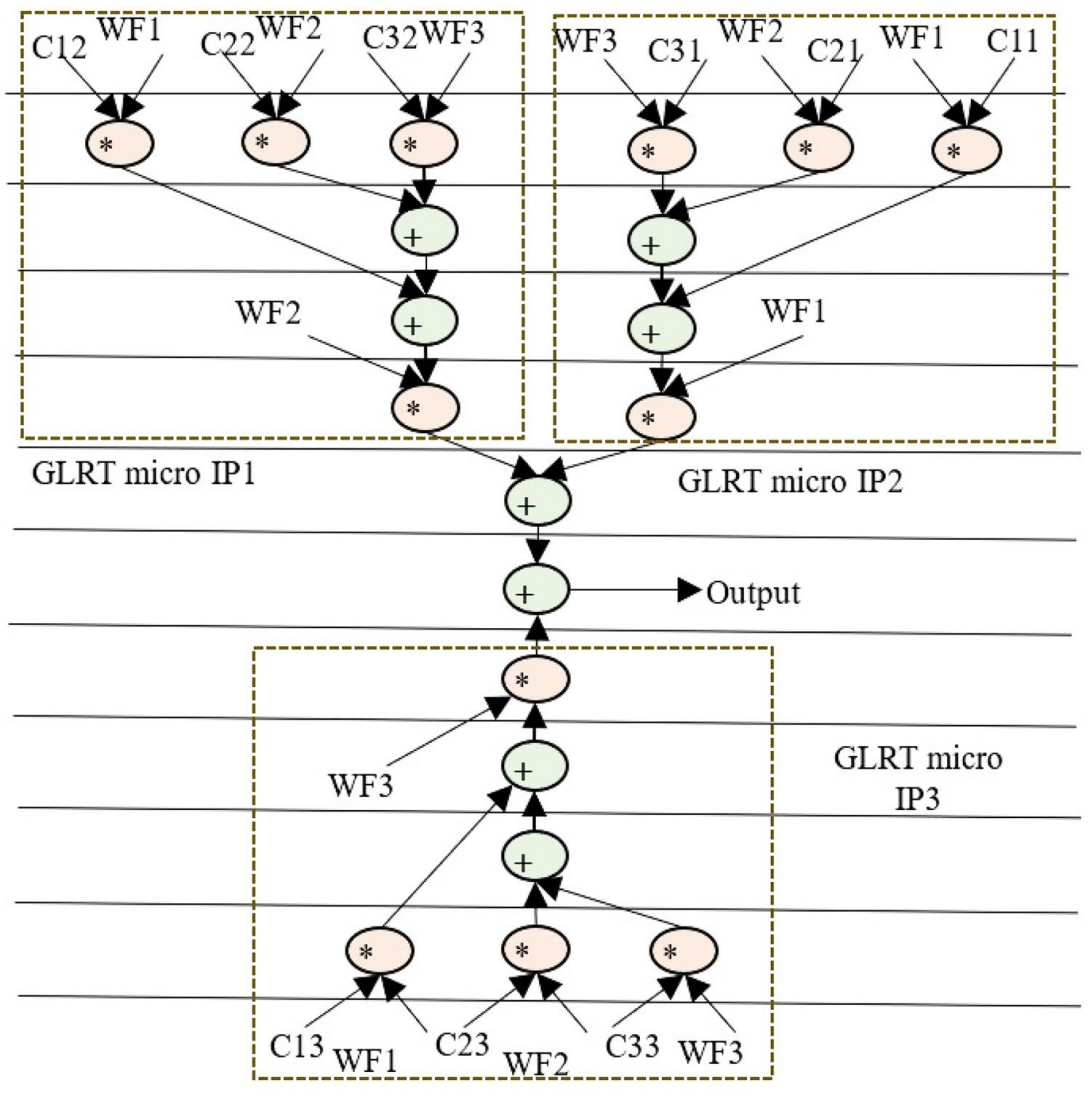
GLRT cascade DFG of proposed macro IP.

The GLRT cascade unit accepts filtered signals as input and evaluates the heart-beat rate of the decomposed wavelet filter bank (WFB) outputs with the threshold function and detects the presence of the QRS complex. The structure uses maximum likelihood manipulation with hardware components that include delays, multipliers, and adders. Figure 2 highlights GLRT cascades 1 and 2 used in an ECG detector. Each GLRT cascade unit comprises of three GLRT micro units. The control data flow graph (CDFG) corresponding to GLRT micro and cascade (extracted from its transfer function^2,3^) is shown in Figs. 3 and 4, respectively. As evident from Fig. 4, the GLRT cascade contains three GLRT micro units. The structure of GLRT cascade stage 1 with primary inputs WF1, WF2, and WF3 (from the filter unit) is replicated in stage 2 with different inputs WF4, WF5, and WF6 (from the filter unit), as shown in Fig. 2. The GLRT micro unit 1 of GLRT cascade stage 1 receives coefficients C12, C22, and C32 for performing computation. Further, the output of GLRT micro unit 1 is fed as input to GLRT micro unit 2 with coefficients C11, C21, and C31. Similarly, the output of GLRT micro unit 2 is fed as input to GLT micro unit 3 with coefficients C13, C23, and C33^3^. Finally, the GLRT cascade unit outputs are summed and compared using a threshold processing unit to differentiate cardiac signals from noise signals.

## Threat model and overview of the proposed methodology

### Threat model

The proposed work is capable of providing detective control (security countermeasure) against pirated IPs including pirated GLRT IP cores before integration into system-on-chip (SoC) of ECG detectors. A pirated GLRT hardware IP integrated in an ECG detector can result in hazardous/fatal consequences for the end user (i.e., patient). Moreover, pirated versions are unreliable as they do not undergo rigorous testing and may contain malicious logic. An attacker realizing the pirated IP of GLRT is usually a competitive rival of the original IP vendor, whose goal is to create fake/pirated IP versions to damage the reputation/goodwill of the original IP vendor while in the process jeopardizing the safety of the end user (patient) using the ECG detector (integrated with unreliable pirated GLRT IP version).

### Motivation

As discussed in the previous sections, it is crucial to design the GLRT unit of the ECG detector as a dedicated reusable hardware IP core. The ability of GLRT to detect QRS complex by performing computation-intensive functions on incoming filter data renders it compatible for designing as a reusable hardware IP core. Further, ensuring the safe and reliable functioning of the designed GLRT hardware IP core is crucial as it facilitates the detection of cardiac signals along with the estimation of critical ECG parametric data. A pirated GLRT hardware IP integrated in an ECG detector can result in hazardous/fatal consequences for the end user (i.e., patient). Moreover, pirated versions are unreliable as they do not undergo rigorous testing and may contain malicious logic. This is because of the involvement of multiple untrustworthy entities such as IP vendors and manufacturing/fabrication houses, posing the peril of unreliability manifesting into piracy based security issues. The presence of malicious logic may also result in erratic behavior of the ECG detector. Further, as discussed previously, the ECG detector is a crucial component of cardiac pacemaker devices; therefore, its reliable functioning is only possible with the integration of authentic (secure) GLRT hardware IP cores. Integration of secure GLRT hardware IP core inside ECG detector enables timely generation of pulse from cardiac pacemaker device. Note: The proposed work aims to provide detective security countermeasure (detective control) against IP piracy (pirated GLRT cores), such that only authentic IP versions are integrated into the chip. The proposed work does not aim to provide preventive control against IP piracy (which is usually performed using locking, metering techniques). In case an attacker pirates the IP and integrates it in his/her chip, then it can be detected/controlled by the authentic IP vendor using the proposed fingerprint biometric detective countermeasure technique.

### Overview

The GLRT micro and GLRT cascade are vital applications used in life critical devices, such as ECG detector and cardiac pacemaker. Therefore, ensuring robust security of GLRT micro and GLRT cascade hardware IPs before integration into SoC of ECG detector/cardiac pacemaker is imperative. Robust security against pirated versions ensures reliable and safe operation of such medical systems, thereby safeguarding the end patient against unreliable behavior. Traditional watermarking mechanisms used for DSP components do not provide adequate security against hardware threats such as tampering, brute force attack, false positives, etc. Therefore, deployment of such conventional watermarking mechanisms do not provide sufficient security countermeasure, especially in the context of life critical medical applications such as GLRT core used in ECG detector/cardiac pacemaker.

Henceforth, in this paper, the proposed methodology presents a security aware design flow to secure micro GLRT micro and GLRT cascade hardware IP core for the ECG detector using the CIG framework of the HLS process and fingerprint biometric to provide enhanced security. The proposed methodology accepts the transfer function of GLRT micro and its cascaded structure, resource configuration (i.e., number of adders and number of multipliers), scheduling algorithm, IP vendor's fingerprint, IP vendor specified truncation length, concatenation rule, and mapping rules. Subsequently, it generates a secure GLRT micro and cascade hardware IP core RTL datapath as output. Initially, the transfer functions corresponding to GLRT micro and its cascaded structure are taken as input and converted into its corresponding control data flow graph (CDFG). Next, the created CDFG is scheduled to generate a scheduled dataflow graph (SDFG) using a scheduling algorithm and IP vendor specified resource constraints. Post SDFG generation, an initial colored interval graph is generated using register allocation information from SDFG. In the next step, a fingerprint biometric based digital template is generated using fingerprint biometric based hardware security methodology. Further, the generated digital template is converted into its equivalent secret hardware security constraints using IP vendor specified mapping rules. The determined secret security constraints are embedded into the initial CIG to generate secret security constraints embedded CIG of GLRT micro and cascaded hardware. The final obtained CIG (containing secret security constraints) is subjected to datapath synthesis to produce a secure GLRT micro and cascade hardware IP core RTL datapath.

## Details of the proposed methodology

### Extraction of proposed GLRT dataflow graph from its transfer function

The GLRT dataflow graph is initially extracted from its corresponding transfer function. The transfer function of GLRT using Mallat's algorithm is adopted from^2,3^:

1 $$Z\left(a\right)={s}^{T}\left(n\right)H({{H}^{T}H)}^{-1}{H}^{T}s(n)$$

where, *s*(*n*) is the input to the filtering unit and *H* is the linear combination matrix of the representative function. Here, *s*^*T*^(*n*) is a 6-by-1 matrix, $H$ is a 1-by-6 matrix, *s*(*n*) is a 1-by-6 matrix and ${(H}^{T}H)$ is a 6-by-6 matrix^34^. The extracted CDFG of GLRT and its cascaded representation are illustrated in Figs. 3 and 4 respectively. WF1, WF2, and WF3 are the outputs of the filtering unit, and C11-C33 is the coefficients of the linear combination matrix H. The extracted CDFG is fed as input to the scheduling allocation and binding block of HLS to generate its corresponding SDFG. The details of the proposed CIG generation from SDFG and fingerprint biometric based hardware security are discussed in the next subsection.

## Proposed secure HLS flow using CIG based encoded fingerprint for secure GLRT micro and cascade hardware IP

**Figure 5:**
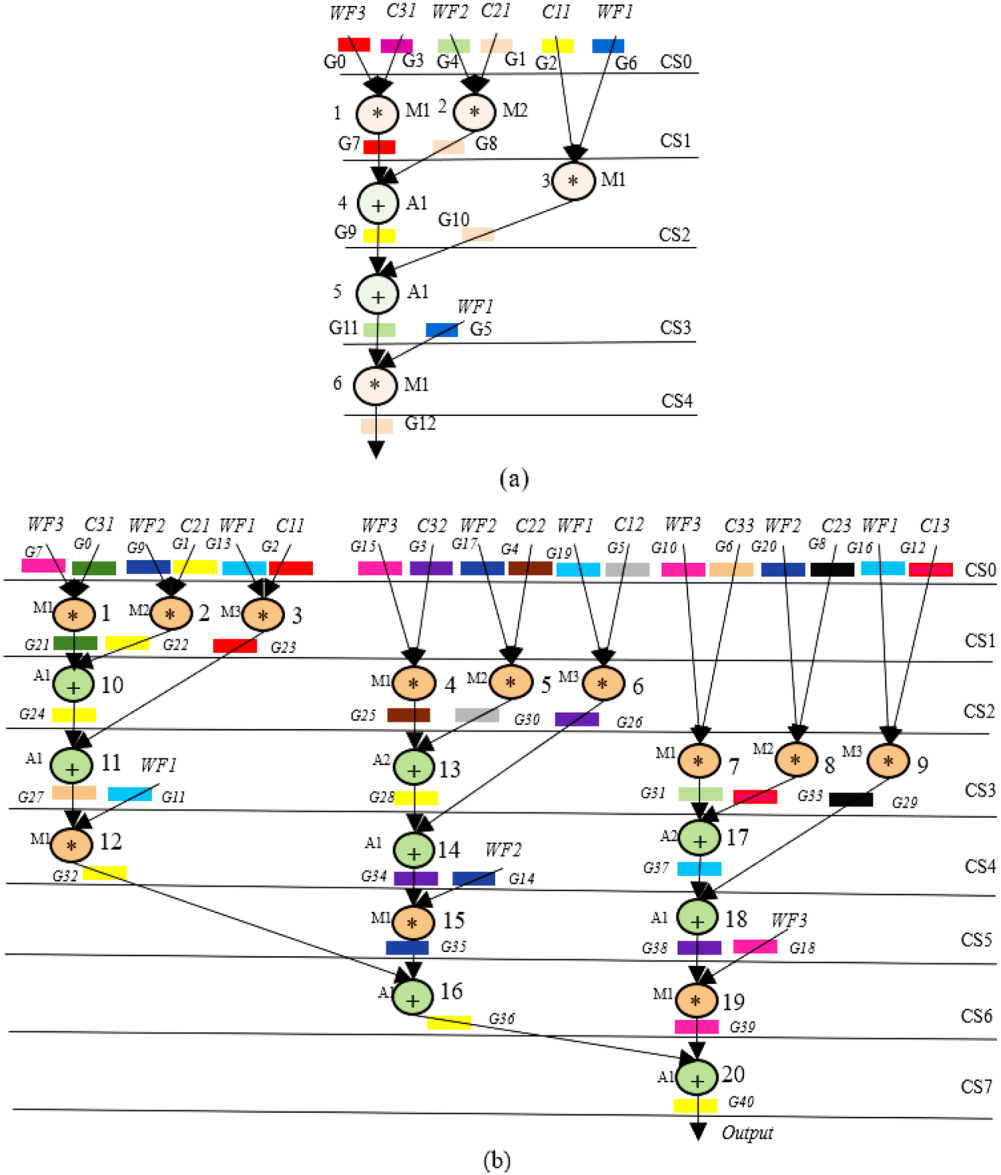
(**a**) SDFG of GLRT micro IP using one adder (+) and two multipliers (*) post embedding fingerprint signature*,* (**b**) SDFG of GLRT cascade macro IP scheduled using three multipliers and two adders post embedding fingerprint.

**Figure 6:**
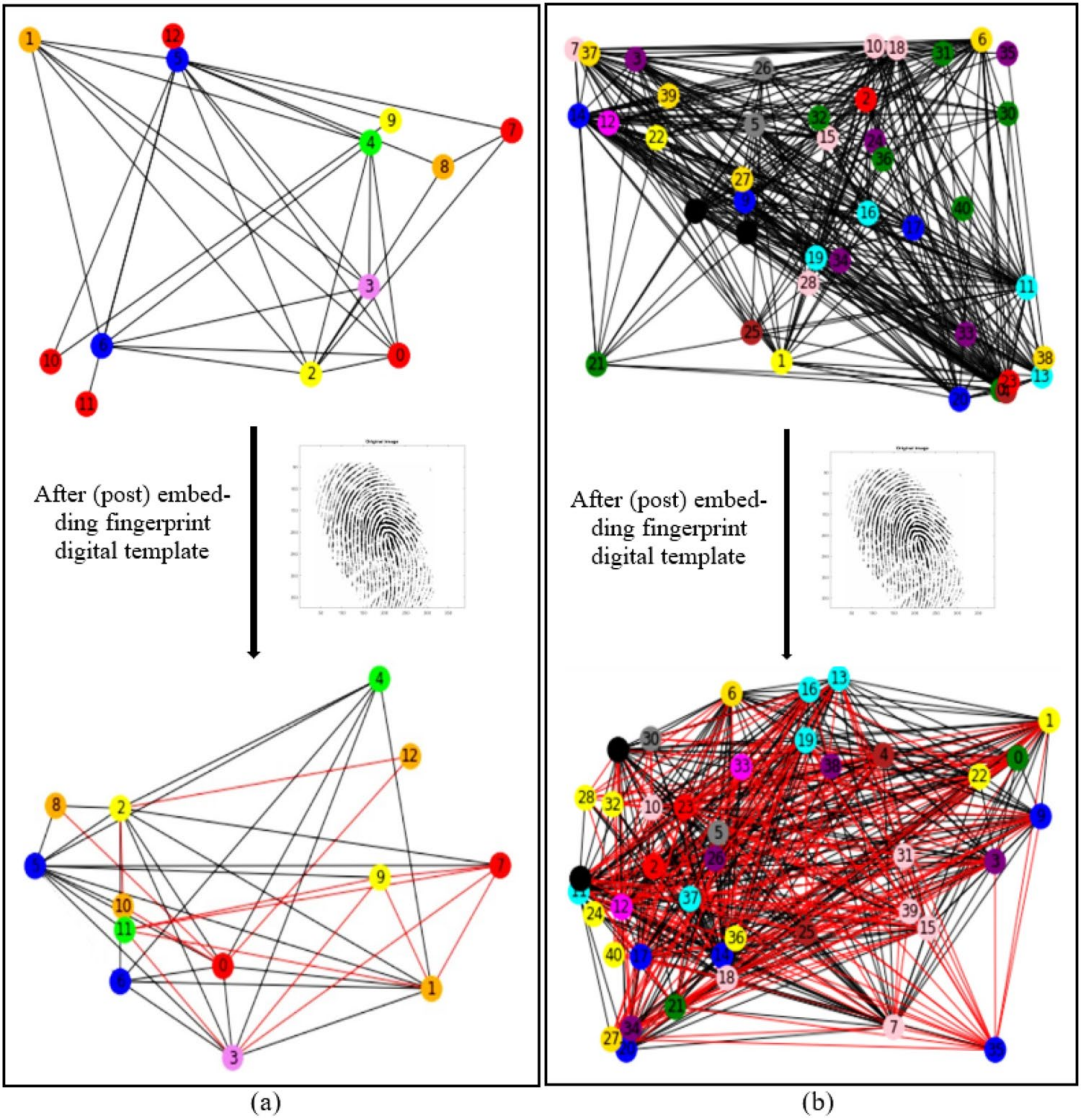
(**a**) CIG (pre and post embedding fingerprint) corresponding to secure GLRT micro IP core, (**b**) CIG (pre and post embedding fingerprint) corresponding to secure GLRT macro IP core.

The proposed secure HLS flow uses a colored interval graph of the HLS framework and fingerprint biometric based hardware security methodology to generate a secure GLRT hardware IP core for ECG detector. As discussed above in the overview subsection, the transfer functions of GLRT micro and GLRT cascade generates its corresponding CDFG. The generated CDFG is fed as the primary input to the scheduling, allocation, and binding (SAB) block of HLS with IP vendor specific resource constraints and scheduling algorithm (such as LIST scheduling) as additional inputs. The final output of the SAB block is an SDFG with registers allocated in different control steps^4^. An SDFG corresponding to micro GLRT and GLRT cascade with register allocations is depicted in Fig. 5a,b, respectively. Registers are storage units that store input, intermediate, and output variable values during the computation of the GLRT unit. Post SDFG creation, an initial CIG is generated using register allocation information of the created GLRT SDFG. CIG is a graphic representation of register allocation information of the GLRT SDFG^4^. The initial CIG (*i.e.,* pre-embedding fingerprint signature based secret security constraints) corresponding to micro GLRT and GLRT cascade are shown in Fig. 6a,b. The obtained initial CIG is used for performing the embedding of fingerprint based secret hardware security constraints. The presence of the IP vendor's fingerprint based covert signature into the design of GLRT hardware IP core guards it against piracy and false claim of IP ownership problems. The generation of fingerprint based covert signature is explained in the following paragraph.

### Generation of fingerprint biometric based covert template/signature

**Figure 7:**
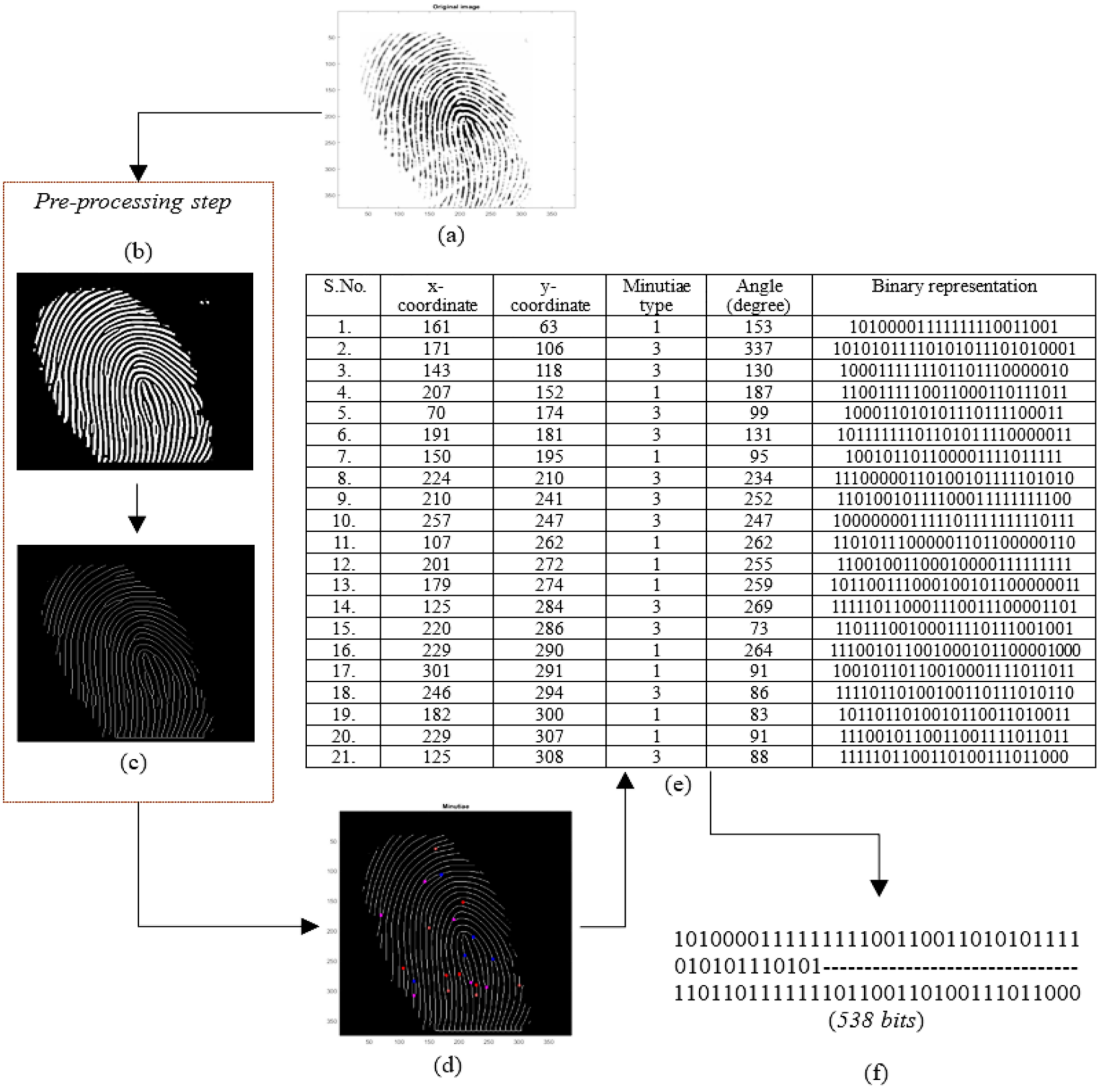
Proposed fingerprint digital template generation process extracted from captured IP vendor’s fingerprint, (**a**) input IP sellers fingerprint image, (**b**) binarized fingerprint image, (**c**) thinned fingerprint image, (**d**) minutiae points generation on fingerprint image, (**e**) details of generated minutiae points parameters, (**f**) generated fingerprint biometric based digital template. The biometric captured is of a real IP vendor entity that is used for further processing of template generation.

We confirm that all experimental protocols were approved by Indian Institute of Technology Indore. A fingerprint biometric based covert signature (*i.e.,* digital template) is generated using IP vendor's biometric fingerprint. Figure 7 illustrates the proposed fingerprint digital template generation process extracted from captured IP vendor's fingerprint. An IP vendor's fingerprint is initially captured using a high-quality optical scanner in a safe, secure, dust-free and trustworthy laboratory environment. The biometric captured is of a real IP vendor entity that is used for further processing of template generation. The other requirements of capturing the fingerprint biometric of IP vendor are (a) the finger used for capturing fingerprint sample should be dirt and grease free, (b) the finger used should be free from injury marks in order to capture all the minutiae points of the fingerprint features (such as ridge angle, bifurcation, etc.), and (c) the optical scanner surface should be clean and dust free in order to capture the fingerprint comprehensively.

**Figure 8:**
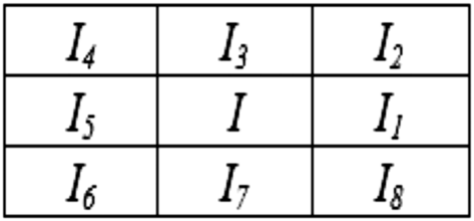
Image matrix representing neighbouring pixels of image pixel *I.*

**Figure 9:**
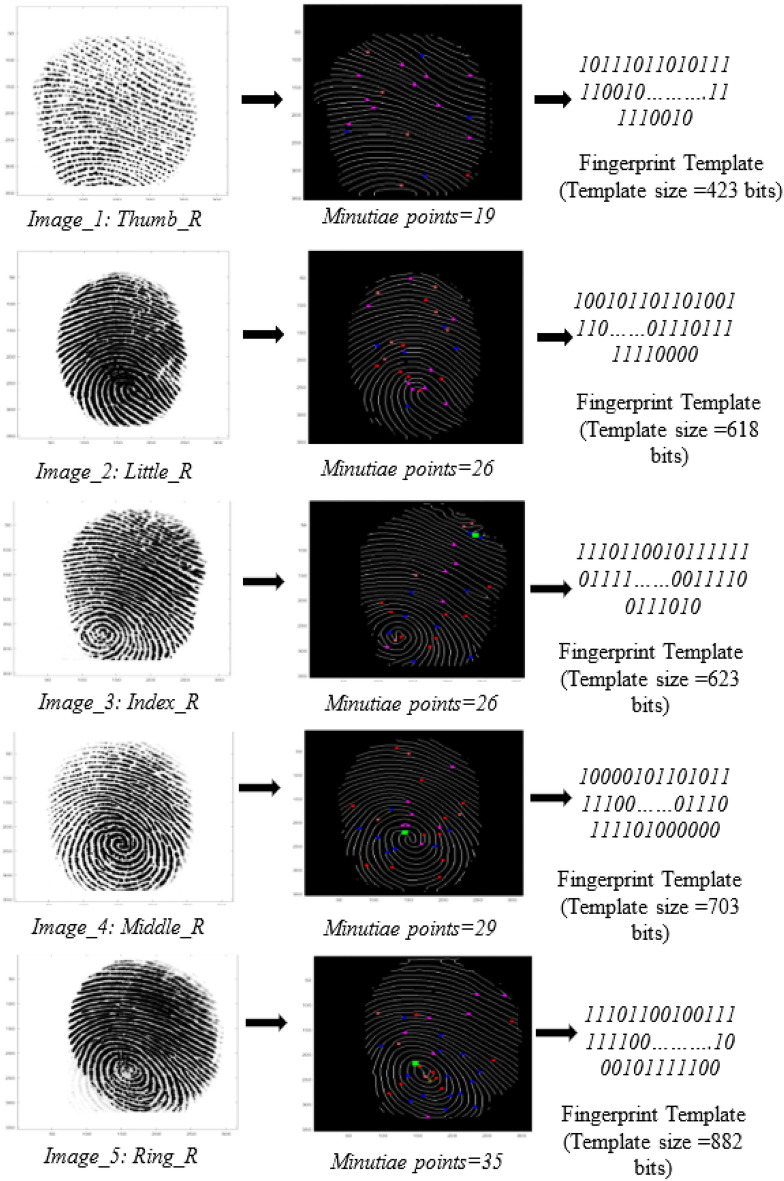
Variation in generated secret fingerprint based constraints for different fingerprints of an IP vendor.

After capturing, the fingerprint is subjected to several preprocessing steps, such as binarization, thinning, and fast Fourier transform (FFT) enhancement. These preprocessing steps are crucial as each step contributes to the generation of an improved fingerprint image, which is used to generate minutiae points. Minutiae points corresponding to the IP vendor's fingerprint are unique and provide uniqueness corresponding to the distinct patterns of the fingerprint features—ridges and valleys. FFT enhancement on the fingerprint image pixels helps in reconnecting the broken ridges, thickening ridges, and separation of parallel ridges. Further, binarization helps in setting the fingerprint image pixels value to either 0 or 255 based on a threshold value. A fingerprint image pixel value lower than the threshold is set to 0, while higher than the threshold, is set to 255. Binarization generates a binary image of the input fingerprint image. Thinning yields a fingerprint image with reduced thickness of ridge line edges, after deletion of the unnecessary pixels. Post-preprocessing, the obtained preprocessed fingerprint image is used to generate minutiae points. The proposed approach employs a crossing number (*C*_*r*_) algorithm to extract the respective minutiae points^35^. Figure 8 shows the neighboring image pixels of image pixel *I*. The crossing number corresponding to a fingerprint image pixel *I* is formulated as^35^:

2 $${C}_{r}=0.5{\sum }_{k=1}^{8}{|I}_{k}-{I}_{k+1}|$$

where *I*_*k*_ is the neighborhood pixel value of pixel *I* (depicted in the above 3 × 3 pixel matrix). A minutiae point is classified into (a) bifurcation and (b) ridge ending. The minutiae point with crossing number 3 is a bifurcation, and crossing number 1 is a ridge ending. Similarly, all minutiae points are generated and classified on the IP vendor's fingerprint. In Fig. 7, red colored dots are ridge ending, and blue color dots are bifurcation type minutiae points. As shown in Fig. 7, twenty-one minutiae points are generated. Post generation of minutiae points, the fingerprint image is placed under IP vendor specified grid size and spacing to extract the crucial parameters, such as x-coordinate, y-coordinate, and ridge angle corresponding to each minutiae point. The ridge angle is a clockwise angle from the horizontal axis. The values corresponding to all four parameters, i.e., x-coordinate, y-coordinate, minutiae type, and ridge angle, are extracted and listed for all generated minutiae points. Further, each decimal value is converted to its binary equivalents and concatenated as per the IP vendor specified concatenation rule. The proposed approach's IP vendor-specific concatenation rule is x-coordinate |+| y-coordinate |+| minutiae type number |+| ridge angle, where |+| is a concatenation operator. Finally, a binary string corresponding to each minutiae point is generated. Subsequently, each binary string is concatenated numerically in ascending order (i.e., minutiae point number) to generate the final fingerprint biometric based digital template (or covert signature). Figure 7 shows the final fingerprint digital template. Moreover, Fig. 9 depicts the variation in generated secret fingerprint based constraints for different fingerprints of an IP vendor.

The generation of secret security constraints using obtained digital fingerprint template and it’s embedding in the initial CIG (pre-embedding) of GLRT hardware IP core is discussed in the next subsection. In the proposed approach, the embedded fingerprint biometric is stored in a secure database in an encrypted format for detection and validation process later. Therefore, recapturing of the fingerprint biometric data is not required for the detection and validation process. During the IP piracy detection process, evasion by an attacker is not possible as he/she is unable to regenerate the fingerprint biometric digital template for embedding into his/her fake versions, thus failing in the hardware IP core authentication process.

## Extraction of proposed GLRT dataflow graph from its transfer function and embedding of security constraints

The obtained digital fingerprint template (comprising of 227 number of zeros and 311 number of ones) is initially converted into secret hardware security constraints using IP vendor specific mapping or embedding rule. The IP vendor specific embedding rule is as follows:

3 $${S}_{0}=\left(G(2s), G(2r)\right)$$

where, 2 s, 2r are whole numbers and (0 ≤ s ≤ 9), (1 ≤ r ≤ 20).

4 $${S}_{1}=\left(G(2s+1), G(2r+1)\right)$$

where (0 ≤ s ≤ 18), (1 ≤ r ≤ 19).

The symbols G(2s) and G(2r) represent the storage variables in the scheduled data flow graph. The limits of *s* and *r* depend on the maximum storage variables used in GLRT SDFG. For bit 0, covert security constraints are generated using *S*_0_, otherwise by using *S*_1_.

The storage variables in the SDFG are sorted in ascending order and stored in a list. Post sorting, the mapping/embedding rule is applied to generate the encoded security constraints using Eqs. (3) and (4), respectively. Therefore, the obtained secret security constraints are as follows: (G0, G2), (G0, G4)–(G0, G40), (G2, G4)–(G2, G40), (G4, G6)–(G4, G40), (G6, G8)–(G6, G40), (G6, G8)–(G6, G40), (G8, G10)–(G18, G40), (G1, G3), (G1, G5)–(G1, 39), (G3, G5)–(G3, G39), (G5, G7)–(G37, G39).

**Figure 10:**
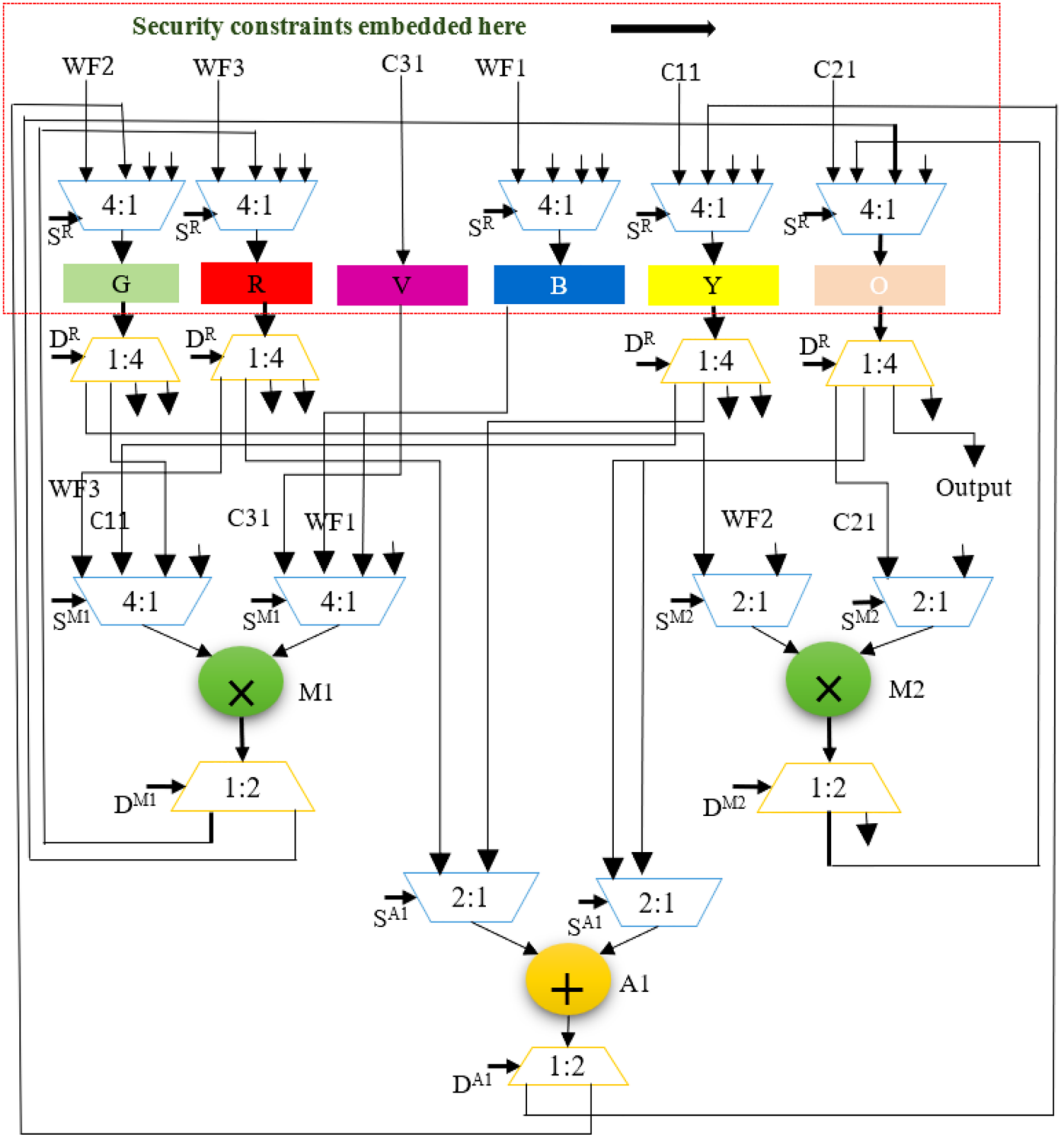
Secure RTL design of GLRT cascade macro IP core with CIG based embedded fingerprint.

**Figure 11:**
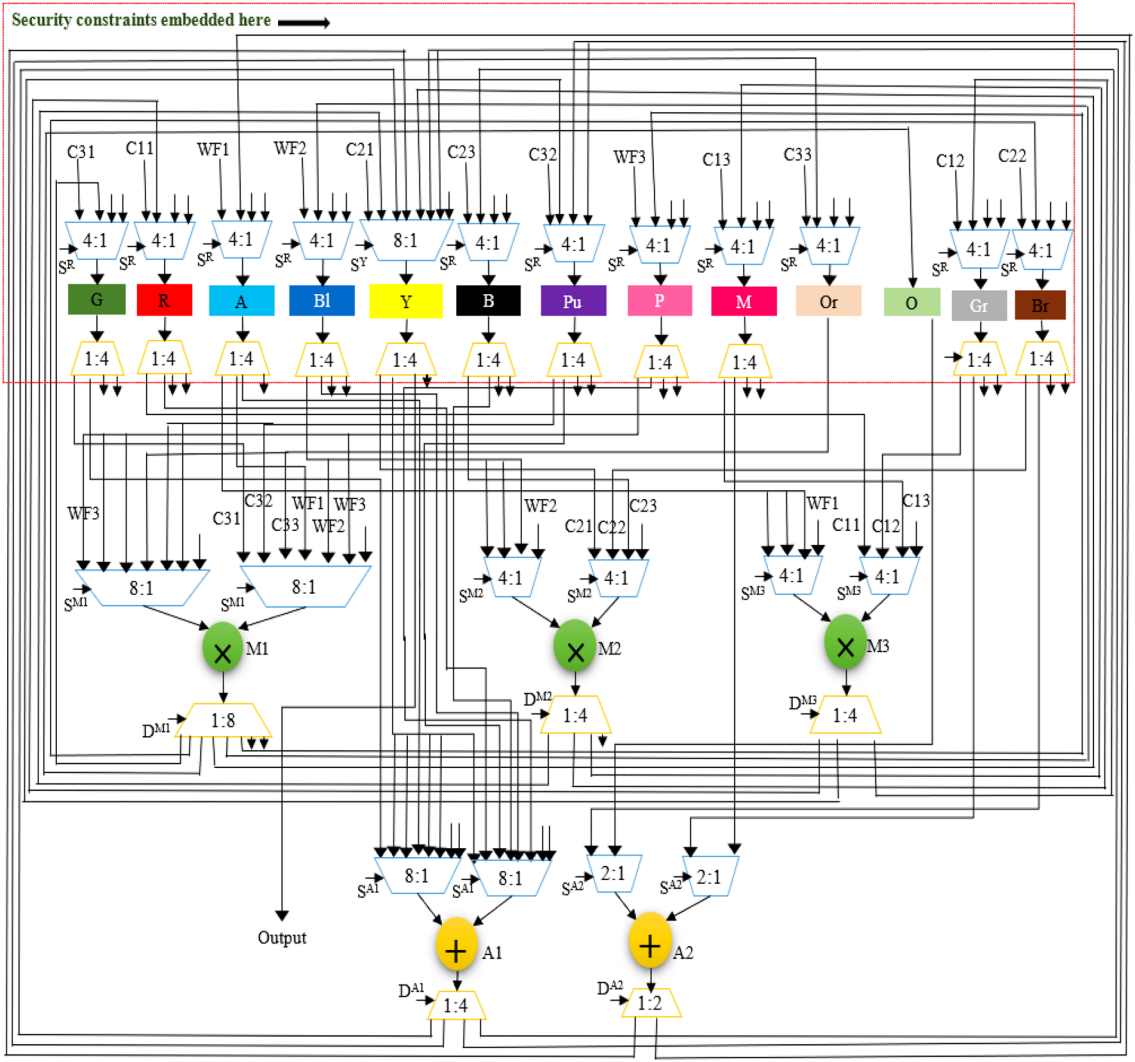
Secure RTL design of GLRT cascade macro IP core with CIG based embedded fingerprint.

Hence, corresponding to 227 number of zeros, 227 security constraints of storage variables are obtained (ranging from (G0, G2)–(G18, G40)). Similarly, corresponding to 311 number of ones, 311 security constraints of storage variables are obtained (ranging from (G1, G3)–(G37, G39)). As evident, these secret security constraints are extracted using the mapping/embedding rule of the IP vendor and is a function of the fingerprint signature obtained. The obtained secret hardware security constraints are embedded into the initial CIG of the micro GLRT and GLRT cascade. No additional changes are made if an edge is already present in the CIG corresponding to incoming secret security constraints. However, in the absence of the edge between storage variables of incoming hardware security constraints, an additional edge in the corresponding CIG is added (depicted in red color in Fig. 6a,b). Post embedding of an additional artificial edge, a color swapping (i.e., local alteration) between registers is performed if the additional embedded edge's storage variables are allocated on the same colored register. The storage variables of embedded security constraints cannot be allocated on the same colored register^4^. Moreover, a new color register is also allocated to resolve the raised conflict if no local alteration is possible. Figure 6a,b depict the final CIG post embedding all determined security constraints corresponding to micro GLRT and GLRT cascade, respectively. As shown in Fig. 6a, storage variables G0 and G12 are allocated on the red color register. However, due to additional artificial edge (G0, G12) (i.e., security constraints), the color of G12 is changed to orange. Similarly, all required alterations are performed post-embedding all security constraints in the CIG of micro GLRT and GLRT cascade. Artificial edges (imposed security constraints) do not alter the functionality of an IP core because these artificial edges are only responsible for local alteration of registers corresponding to storage variable assignment. These imposed security constraints do not affect the dataflow connectivity of the SDFG corresponding to the IP core functionality. Therefore, due to embedding of the security constraints, the functional units and its interconnectivity remain unaffected (only the register sharing is locally altered). Further, Fig. 5a,b illustrate the SDFG of micro GLRT and GLRT cascade post-embedding hardware security constraints. Moreover, Figs. 10 and 11 depict the secure RTL datapath corresponding to the micro GLRT hardware IP core and GLRT cascade macro hardware IP core for the ECG detector. The final generated RTL of GLRT hardware IP core contains security constraints in the form of altered register colors. The hardware design of GLRT micro IP comprises of two multiplier units and one adder unit as functional units, six registers (G, R, Y, V, B, Y, O) as storage elements, seven 4:1 multiplexer units, four 2:1 multiplexer units, three 1:2 demultiplexer units and 1:4 demultiplexer units, all synchronized/timed through a centralized controller unit [implemented as a finite state machine (FSM)]. The control signals responsible for providing the timing to the datapath of the micro IP are as follows: S^R^, S^M1^, S^M2^, S^A1^ for activating/deactivating the 4:1 multiplexers and 2:1 multiplexers respectively; D^R^, D^M1^, D^M2^ and D^A1^ control signal for providing deselect to the 1:4 demultiplexers and 1:2 demultiplexers respectively; Register strobe signals for activating the six registers that are responsible for storing input data, intermediate outputs and final output specified through storage variables G0 to G12 (as per the SDFG of micro IP shown in Fig. 5a). The fingerprint security constraints are accommodated into the portion marked in red boundary (after register sharing are locally altered). The controller (FSM) consumes five control steps during scheduling for computing the final output of the micro GLRP IP. The datapath of the micro IP and its controller are implemented using very high-speed integrated circuit hardware description language (VHDL) for synthesis/simulation in Intel Quartus tool version 13.0sp1. The hardware components of the datapath IP were each implemented as VHDL, followed by creating a top-level entity containing the port-mapped VHDL file comprising of the interconnected datapath components. The top-level design along with its components and controller design were executed for functional validation. Post-implementation, 862 logic elements are utilized for micro IP GLRT with respect to Cyclone IV GX field-programmable gate array (FPGA).

Similarly, the hardware design of GLRT cascade macro IP comprises of three multipliers and two adders as functional units, thirteen registers (G, R, A, B1, Y, B, Pu, P, M, Or, O, Gr, Br) as storage elements, fifteen 4:1 multiplexers, three 8:1 multiplexers, two 2:1 multiplexers, fourteen 1:4 demultiplexer units, one 1:8 demultiplexer unit and one demultiplexer unit, all synchronized/timed through a centralized controller unit (implemented as a finite state machine). The control signals responsible for providing the timing to the datapath of the cascade macro IP are as follows: S^R^, S^M1^, S^M2^, S^M3^, S^A1^, S^A2^ for activating/deactivating the 4:1 multiplexers, 8:1 multiplexers and 2:1 multiplexers respectively; D^R^, D^M1^, D^M2^, D^M3^, D^A2^ and D^A1^ control signal for providing deselect to the 1:4 demultiplexers, 1:8 demultiplexers and 1:2 demultiplexers respectively. Register strobe signals for activating the thirteen registers that are responsible for storing input data, intermediate outputs and final output specified through storage variables G0 to G40 (as per the SDFG of micro IP shown in Fig. 5a). The fingerprint security constraints are accommodated into the portion marked in red boundary (after register sharing are locally altered). The controller (FSM) consumes eight control steps during scheduling for computing the final output of the micro GLRP IP. The datapath of the cascade macro IP and its controller are implemented using hardware description language (VHDL) for synthesis/simulation in Intel Quartus tool. Post-implementation, 2021 logic elements are utilized for macro GLRT cascade IP with respect to Cyclone IV GX FPGA.

## Detection of pirated hardware IP core of GLRT for ECG detector

The presence of fingerprint biometric based digital evidence helps in making a clear distinction between authentic and fake (i.e., pirated) versions of GLRT hardware IP cores. While conducting piracy detection, the security constraints corresponding to the IP vendor's fingerprint template are initially regenerated. The regenerated information is matched with the register allocation information of GLRT hardware IP's RTL datapath under test. The authentic version will exhibit a complete matching of security constraints; otherwise, the version is considered pirated.

It is crucial to protect the designed GLRT hardware IP core for the ECG detector from an adversary's false claim of IP ownership problem. An adversary located at an untrustworthy offshore design or fabrication house can falsely claim hardware IP ownership right. The IP vendor's digital fingerprint template embedded in the design (i.e., CIG) of GLRT hardware IP core safeguards it from the adversary's false claim of IP ownership. Authentic IP vendor can easily nullify the false claim of IP ownership by matching the embedded digital fingerprint constraints (in the GLRT RTL IP under test) with the original one (minutiae points pre-stored in a secure database in an encrypted format or can be regenerated as explained above in the previous subsection). In case of complete matching, ownership is awarded to the original IP vendor. In the proposed approach, the embedded fingerprint biometric is stored in a secure database in an encrypted format for detection and validation process later. Therefore, recapturing of the fingerprint biometric data is not required for the detection and validation process.

## Results and analysis

The experimental assessment of the proposed secure GLRT hardware IP core design for the ECG detector has been performed on a system with a 2.30 GHz processor and 4 GB RAM. A 15 nm technology scale based on the NanGate library^36^ is used in the proposed approach to evaluate design area and latency corresponding to secure GLRT hardware IP core. The proposed design in this paper is a simulated version of the secure GLRT hardware IP at register transfer level (RTL). In case fabricated version of the design RTL is intended using the layout level information, standard CAD tool based design synthesis steps at lower levels can be employed to generate the layout level representation of the designed secure GLRT hardware IP (at RTL). However, building the layout design of the secure GLRT hardware IP is not the target of the work and beyond the scope of this current paper.

## Security assessment in terms of probability of coincidence and tamper tolerance

Embedding the IP vendor's digital fingerprint template provides robust security to the designed GLRT hardware IP core of the ECG detector. This is because of the following reasons: (a) IP vendor's fingerprint digital template facilitates the integration of a unique natural identity with the design synthesis flow that increases the robustness of the proposed approach against IP piracy and false claim of IP ownership, and (b) the inclusion of several IP vendor specific parameters and rules such as concatenation rule, mapping rule, truncation length, etc. hinders the adversary from exactly regenerating the digital fingerprint template. The security analysis of the proposed secure GLRT hardware IP core is performed using established security metrics in the literature^4,10,15,37,38^ such as (a) probability of coincidence (false positive), (b) tamper tolerance.

The probability of coincidence (*C*_*i*_) security metric reports the likelihood of coincidently locating the same security constraints (i.e., same fingerprint digital template) in an unsecured (or non-watermarked) GLRT hardware IP core. It is a measure of false positive and also indicates the probability of watermark collision of authentic security constraints being coincidently present in an unsecured design version^39^. Therefore, a lower value of *C*_*i*_ is desirable. *C*_*i*_ is formulated as^15,40,41^ and^17,42,43^:

5 $${C}_{i}={(1-\frac{1}{z})}^{c}$$

where *c* is the embedded fingerprint security constraint and *z* is the total register required post-scheduling pre-embedding security constraints. A lower value of *C*_*i*_ indicates more robust security as the likelihood of detecting the same digital template decreases with the decrease in *C*_*i*_ value. Tables 2 and 3 report the comparison of *C*_*i*_ between the proposed secure GLRT cascade hardware IP with embedded fingerprint and secure GLRT cascade hardware IP with facial biometric^44^, digital signature^19^, encrypted signature^20^ and hardware watermarking^15^. The proposed secure GLRT cascade hardware IP core with embedded fingerprint surpasses^15,19,20^, and^44^, as clear from Tables 2 and 3. The determination of the larger number of security constraints in the proposed approach helps in achieving a lower value of *C*_*i*_ than^15,19,20^, and^44^. Embedding a larger number of security constraints (i.e., the presence of greater digital evidence in the design) increases the attacker's effort to locate the same security constraints in an unsecured GLRT hardware IP design.

**Table 2:** Comparison of probability of coincidence (*C*_*i*_) between the proposed fingerprint embedded secure GLRT cascade IP with facial biometric^44^ embedded IP design and digital signature embedded IP design^19^.

Proposed secure GLRT IP with fingerprint		Design with facial constraints^44^		Design with digital signature^19^	
Security constraints	*C*_*i*_	Security constraints	*C*_*i*_	Security constraints	*C*_*i*_
250	3.57E−10	16	2.48E−01	16	2.48E−01
275	4.05E−11	32	6.17E−02	32	7.71E−02
300	4.60E−12	64	3.81E−03	64	3.81E−03
346	8.41E−14	81	8.69E−04	128	1.45E−05

**Table 3:** Comparison of probability of coincidence (*C*_*i*_) between the proposed fingerprint embedded secure GLRT cascade IP with encrypted signature embedded IP design^20^ and hardware watermarking embedded IP design^15^.

Proposed secure GLRT IP with fingerprint		Design with encrypted signature^20^		Design with watermark^15^	
Security constraints	*C*_*i*_	Security constraints	*C*_*i*_	Security constraints	*C*_*i*_
250	3.57E−10	32	6.17E−02	32	6.17E−02
275	4.05E−11	64	3.81E−03	64	3.81E−03
300	4.60E−12	128	1.45E−05	128	1.45E−05
346	8.41E−14	160	8.99E−07	240	8.52E−10

Further, the second security metric, i.e., tamper tolerance (*T*_*i*_), depicts the adversary's effort in employing brute force attack to guess/tamper the exact embedded signature combination. Using this, an attacker may attempt to evade the IP piracy detection process. The higher the *T*_*i*_, the greater the effort required by an adversary. The *T*_*i*_ is formulated as^15,40,41^ and^17^:

6 $${T}_{i}={e}^{c}$$

where *c* is the embedded fingerprint security constraint and *e* is the total encoding variables required to perform mapping of the template to security constraints. Tables 4 and 5 show the comparison of *T*_*i*_ between the proposed secure GLRT cascade hardware IP with embedded fingerprint and secure GLRT cascade hardware IP with facial biometric^44^, digital signature^19^, encrypted signature^20^ and hardware watermarking^15^. The proposed approach supersedes^15,19,20^, and^44^, as clear from Tables 4 and 5, due to the determination of larger security constraints. A higher value of *T*_*i*_ signifies a larger signature space because of greater signature combinations. This makes it significantly harder by increasing the attacker's effort/time to guess the exact embedded signature combination from the larger signature space.

**Table 4:** Comparison of tamper tolerance (*T*_*i*_) between the proposed fingerprint embedded secure GLRT cascade IP with facial biometric^44^ embedded IP design and digital signature embedded IP design^19^.

Proposed secure GLRT IP with fingerprint		Design with facial constraints^44^		Design with digital signature^19^	
Security constraints	*T*_*i*_	Security constraints	*T*_*i*_	Security constraints	*T*_*i*_
250	1.80E + 75	16	6.55E + 04	16	6.55E + 04
275	6.07E + 82	32	4.29E + 09	32	4.29E + 09
300	2.03E + 90	64	1.84E + 19	64	1.84E + 19
346	1.43E + 104	81	2.41E + 24	128	3.40E + 38

**Table 5:** Comparison of tamper tolerance (*T*_*i*_) between the proposed fingerprint embedded secure GLRT cascade IP with encrypted signature embedded IP design^20^ and hardware watermarking embedded IP design^15^.

Proposed secure GLRT IP with fingerprint		Design with encrypted signature^20^		Design with watermark^15^	
Security constraints	*T*_*i*_	Security constraints	*T*_*i*_	Security constraints	*T*_*i*_
250	1.80E + 75	32	6.17E−02	32	6.17E−02
275	6.07E + 82	64	3.81E−03	64	3.81E−03
300	2.03E + 90	128	1.45E−05	128	1.45E−05
346	1.43E + 104	160	8.99E−07	240	8.52E−10

### Design cost (*D*_*c*_) and power evaluation

**Figure 12:**
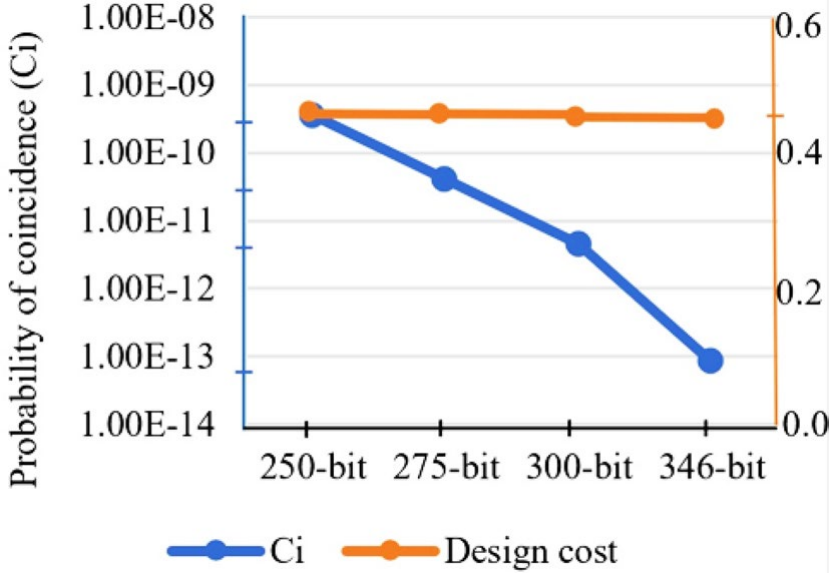
Design cost vs probability of coincidence trade-off for proposed secure GLRT cascade for varying fingerprint signature sizes.

The design cost assessment of the proposed secure GLRT hardware IP core is performed using Eq. (7). Table 6 reports the design latency, area, and IP vendor specified resource configuration of the proposed secure GLRT hardware IP for the ECG detector before and after embedding the fingerprint template. As evident from Table 6, the proposed secure GLRT hardware IP core with fingerprint biometric provides robust security at zero design cost overhead (i.e., no extra registers are required). As evident from Table 7, the power overhead of the proposed approach is 0%, as the post fingerprint embedded design does not incur any extra functional units or registers. Table 7 also reports the leakage power value of pre-embedded and post fingerprint embedded GLRT IP core. Therefore, the proposed secure GLRT IP core produces reliable designs as low-power designs result into lesser heat dissipation. Further, Fig. 12 highlights the design cost *vs.* probability of coincidence tradeoff for the proposed secure GLRT cascade for varying fingerprint signature sizes. The value of *C*_*i*_ decreases with an increase in signature size at constant value of design cost.

7 $${D}_{c}=\left({e}_{1}\left(\frac{{L}_{T}}{{L}_{max}} \right)\right)+ \left({e}_{2}\left(\frac{{A}_{R}}{{A}_{max}}\right)\right)$$

where, *e*_*1*_ = *e*_*2*_ = 0.5 for giving equal weightage to design latency and area, *L*_*T*_ and *A*_*R*_ are design latency and area corresponding to GLRT hardware IP. Further, *L*_*max*_ and *A*_*max*_ are their corresponding maximum latency and area, respectively.

**Table 6:** Design latency, area, and resource configuration of proposed secure GLRT IP before and after embedding fingerprint signature.

Application	Resource configuration	Unsecured design (before fingerprint embedding)		Proposed fingerprint embedded secure design	
		Design area (µm^2^)	Design latency (ps)	Design area (µm^2^)	Design latency (ps)
GLRT cascade hardware IP core	2(+), 3(*)	273.67	1656.07	273.67	1656.07

**Table 7:** Design cost, leakage power, register count and resource configuration of proposed secure GLRT hardware IP before and after embedding fingerprint signature.

Application	Resource configuration and registers	Unsecured design (before fingerprint embedding)		Proposed fingerprint embedded secure design	
		Design cost	Leakage power	Design cost	Leakage power
GLRT cascade hardware IP core	2(+), 3(*), and 13 registers	0.43	8.57 *μw*	0.43	8.57 *μw*

### Security analysis against possible attacks

#### Security against forgery and spoofing attack

Forgery and spoofing are not feasible in the case of the proposed approach. This is because the biometric fingerprint minutiae points are pre-stored in an encrypted format in a safe database for validation/detection later. Any attacker endeavoring to forge the stored encrypted biometric fingerprint template would be unsuccessful in using it since he/she does not have the knowledge of the advanced encryption standard (AES) private key needed for decryption. Furthermore, a spoofing attack is not applicable in the case of IP piracy detection. This is because the attacker's goal is to evade/escape IP piracy detection by re-moving/tampering with the original embedded secret signature (security constraints). However, an attacker may attempt to launch spoofing to falsely claim IP ownership, which is not possible as spoofing of encrypted biometric fingerprint template requires forgery of the pre-stored encrypted biometric fingerprint template, which is not useful until an attacker is capable of successfully decrypting the encrypted template using the AES private key. Besides, an attacker also needs to decode the following security parameters: (a) the number of features used in each template for fingerprint, (b) number of minutiae points and their exact 4-dimensional coordinates used for template regeneration, and (c) concatenation order of minutiae points used for regenerating the fingerprint template, for performing successful and accurate spoofing to falsely claim IP ownership. Therefore, forgery and spoofing attack is not possible in the proposed approach.

#### Security against side channel attack (SCA) and machine learning (ML)-based attack

The proposed security methodology stands strong against SCA and ML-based attacks, in contrast to PUF-based techniques. This resilience is attributed to the fact that the proposed security approach incurs a zero impact on the overall design cost of GLRT IP (as elaborated in “Design cost (D_c_) and power evaluation” and Table 5). Hence, the secret biometric fingerprint watermark embedded design does not leak significant side-channel information (such as delay, power, etc.). As previously mentioned, in the proposed approach, the biometric fingerprint watermark constraints (digital evidence) are embedded solely by locally modifying the register assignments (through swapping). Consequently, there is no noticeable impact on side channel parameters from an attacker's perspective. Furthermore, ML attacks are not applicable to a design with an embedded watermark (in case of proposed approach), as it does not rely on challenge-response pairs, which are prime targets for adversarial/modelling attacks, contrary to PUF-based systems.

#### Security against brute-force attack (tamper tolerance)

An attacker may attempt to perform a brute-force attack to remove/tamper the original embedded secret watermark (fingerprint security constraints). Tamper tolerance measures security in terms of the brute force attempts by adversaries to tamper the design or guess the exact signature combination. A higher *T*_*Z*_ value is desirable as it indicates a larger signature space, resulting in huge possible signature combinations. A higher *T*_*Z*_ value increases the complexity for attackers in their attempts to discover the exact watermark signature combination (security constraints). In the case of the proposed approach, *T*_*Z*_ is extremely high when launching a brute-force attack. Therefore, removing the embedded biometric fingerprint based watermark (security constraints) of the proposed approach is highly challenging. “Security assessment in terms of probability of coincidence and tamper tolerance” (Tables 4, 5 highlights the strength of the proposed approach in terms of tamper tolerance.

#### Security against ghost signature search attack and false positive/watermark collision (probability of coincidence)

The credibility of the embedded secret watermark should be seamlessly detectable for the evidence of authorship. No third party (i.e., other than the IP owner) should be able to claim the watermark by chance (in order words watermark collision should be as low as possible). The probability of coincidence serves as a metric to assess the likelihood of coincidently detecting the exact security constraints within an unsecured IP design (false positive). The likelihood of a successful ghost signature search attack is the same as the probability of coincidence. Thus, a lower *C*_*i*_ value signifies more robust security and stronger credibility, indicating higher level of security. “Security assessment in terms of probability of coincidence and tamper tolerance” (Tables 2 and 3) highlights the strength of the proposed approach in terms of probability of coincidence.

## Conclusion

This paper discussed a novel secure aware design methodology for GLRT cascade hardware IP core integrated with CIG based fingerprint biometric. Designing a secure GLRT hardware IP core with embedded fingerprint ensures successful distinction from its pirated version (using robust detection process) and enables integration of its authentication version only in ECG detector unit, which is highly essential for the safety of the end patient. This is because as discussed in the paper, integration of pirated versions in ECG detector units can be hazardous for the end safety of the patient. Therefore, through the proposed approach, reliable and secure functioning of ECG detectors is possible which is highly crucial to correctly analyze a patient's heart condition.

## Data Availability

The datasets used and/or analyzed during the current study is available from the corresponding author on reasonable request.
